# Recent advancements in mass spectrometry–based tools to investigate newly synthesized proteins

**DOI:** 10.1016/j.cbpa.2021.07.001

**Published:** 2022-02

**Authors:** Wouter van Bergen, Albert J.R. Heck, Marc P. Baggelaar

**Affiliations:** 1 Biomolecular Mass Spectrometry and Proteomics, Bijvoet Center for Biomolecular Research and Utrecht Institute for Pharmaceutical Sciences, University of Utrecht, Padualaan 8, Utrecht, 3584 CH, the Netherlands; 2 Netherlands Proteomics Center, Padualaan 8, Utrecht, 3584 CH, the Netherlands

**Keywords:** Newly synthesized proteins, Protein synthesis, Protein dynamics, Mass spectrometry, Proteomics, Bio-orthogonal noncanonical amino acid tagging (BONCAT), Puromycin, Stable isotope labeling by amino acids in cell culture (SILAC), AHA, *L*-azidohomoalanine, ANL, *L*-azidonorleucine, BONCAT, Bio-orthogonal non-canonical amino acid tagging, HPG, *L*-homopropargylglycine, iTRAQ, Isobaric tag for relative and absolute quantitation, LC, Liquid chromatography, LPS, Lipopolysaccharide, mePROD, Multiplexed enhanced protein dynamics, MITNCAT, Multiplex isobaric tagging/non-canonical amino acid tagging multiplex, mPDP, Multiplexed proteome dynamics profiling, mRNA, Messenger ribonucleic acid, MS, Mass spectrometry, NCAA, Non-canonical amino acid, NSP, Newly synthesized protein, OPP, O-propargyl-puromycin, pSILAC, pulsed SILAC, PUNCH–P, Puromycin-associated nascent chain proteomics, QuaNCAT, Quantitative non-canonical amino acid tagging, RNA, Ribonucleic acid, SILAC, Stable isotope labeling by amino acids in cell culture, TMT, Tandem mass tag, TNF-α, Tumor necrosis factor alpha, tRNA, Transfer-ribonucleic acid

## Abstract

Tight regulation of protein translation drives the proteome to undergo changes under influence of extracellular or intracellular signals. Despite mass spectrometry–based proteomics being an excellent method to study differences in protein abundance in complex proteomes, analyzing minute or rapid changes in protein synthesis and abundance remains challenging. Therefore, several dedicated techniques to directly detect and quantify newly synthesized proteins have been developed, notably puromycin-based, bio-orthogonal noncanonical amino acid tagging–based, and stable isotope labeling by amino acids in cell culture–based methods, combined with mass spectrometry. These techniques have enabled the investigation of perturbations, stress, or stimuli on protein synthesis. Improvements of these methods are still necessary to overcome various remaining limitations. Recent improvements include enhanced enrichment approaches and combinations with various stable isotope labeling techniques, which allow for more accurate analysis and comparison between conditions on shorter timeframes and in more challenging systems. Here, we aim to review the current state in this field.

## Graphical abstract

**Figure:**
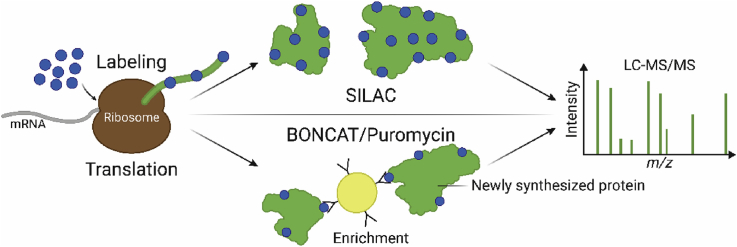

## Introduction

**Figure 1:**
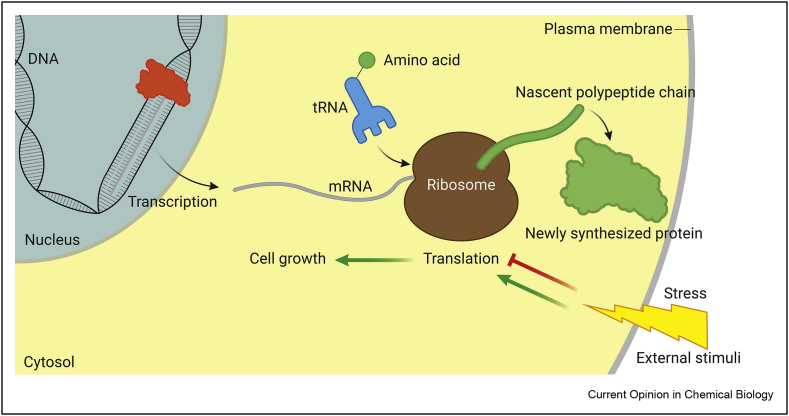
**Protein translation overview**. mRNA is transcribed from DNA in the nucleus before it is transported to the cytosol where it is translated into protein by ribosomes. tRNA molecules serve as the link between mRNA and the ribosome by presentation of mRNA-encoded amino acids to the ribosome for elongation of the nascent polypeptide chain, generating a newly synthesized protein.

Protein translation is the gatekeeper between the genome and the proteome by serving as the final regulatory layer before gene expression. This multistep process comprises initiation, elongation, termination, and ribosome recycling. Protein synthesis is a fundamental process that is strongly connected to cell growth (Figure 1). Protein synthesis is generally upregulated in tumor cells, and consequently, inhibition of protein synthesis is an attractive strategy for cancer treatment [1,2].

In addition to proteome expansion to facilitate cell growth, spatiotemporal control of the translational machinery on protein synthesis by translation of newly transcribed mRNA or pre-existing mRNA pools orchestrates rapid adaptations to environmental cues and internal signals. Thereby, protein synthesis underlies many crucial physiological processes such as learning and memory formation, T cell activation, and inflammation [3].

Although the importance of protein synthesis is well recognized, quantitative analysis of protein synthesis has proven to be challenging. The rate of protein synthesis can be indirectly inferred from mRNA levels measured by RNA-sequencing or ribosome profiling. However, mRNA levels show limited correlation to protein synthesis because translation is, as the final regulatory layer of the proteome, a highly and tightly regulated process. This allows the cell to rapidly synthesize specific proteins under stress or other extracellular or intracellular influences [4,5]. Direct analysis of synthesis at the protein level is therefore indispensable to investigate alterations in the proteome over time or in response to stimuli. Here, we review recent progress in the development of mass spectrometry (MS)–based tools to investigate, at a proteome-wide level, protein synthesis.

### Mass spectrometry–based proteomics

**Figure 2:**
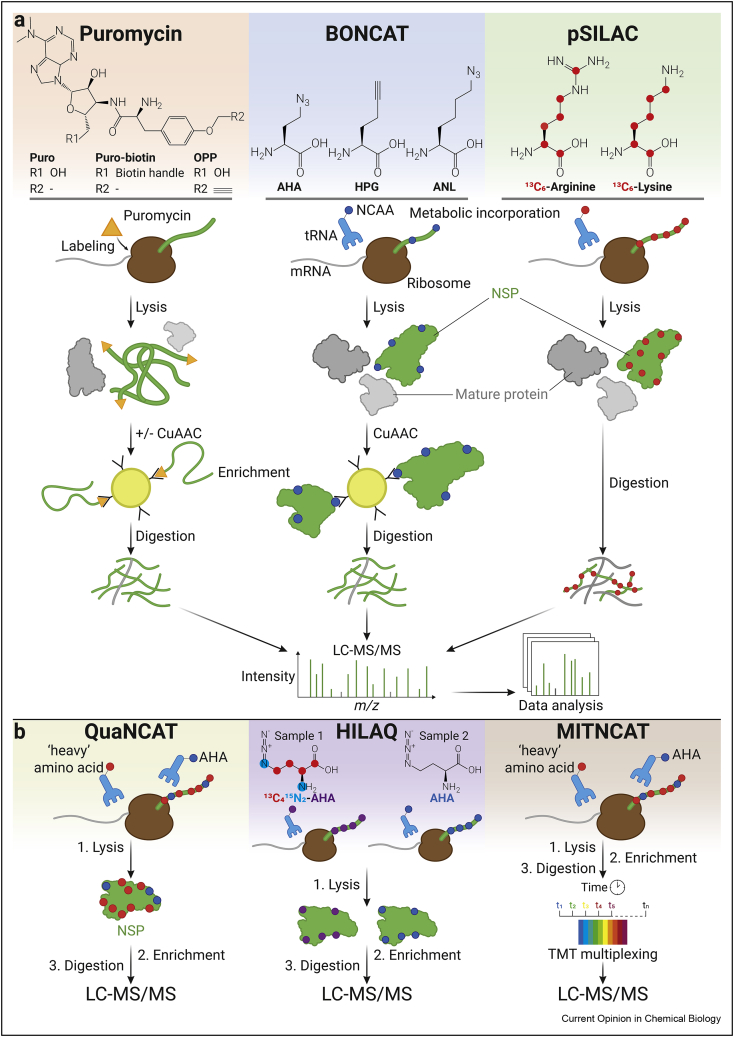
**Mass spectrometry–based methods for analysis of newly synthesized proteins**. (**a**) Currently used MS-based methods to analyze NSPs can be categorized into three main strategies. 1. The puromycin-based strategy relies on the aminonucleoside antibiotic, puromycin (Puro), which inhibits protein synthesis and couples to the C-terminus of nascent polypeptide chains (NPCs). Biotinylated and alkynylated variants of puromycin enable targeted enrichment and measurement of NPCs by LC-MS; 2. BONCAT-based methods rely on the methionine surrogates AHA, HPG, or ANL to enrich for NSPs. After their metabolic incorporation in NSPs, a copper (I)-catalyzed alkyne–azide cycloaddition (CuAAC) can be used to functionalize labeled NSPs with affinity handles, such as biotin, to enable enrichment; 3. SILAC relies on metabolic labeling of NSPs with isotopically labeled amino acids. The strategy does not contain an enrichment step, but NSPs can be identified by LC-MS/MS detection of the isotopically labeled amino acids. (**b**) Combinations of BONCAT with other quantitative techniques to enhance the accuracy and temporal resolution of NSP analysis. QuaNCAT combines BONCAT with pSILAC, to discriminate between *bona fide* NSPs and false positives. Heavy isotope-labeled AHA quantification (HILAQ) uses stable isotope-labeled AHA and nonlabeled AHA for relative quantification of two different conditions. MITNCAT (multiplex isobaric tagging/noncanonical amino acid tagging) combines QuaNCAT and TMT multiplexing to reduce labeling time and allows detection of small changes in protein synthesis in short timeframes.

Continuous advances in the capabilities and performance of high-resolution mass spectrometers allow the study of the proteome with increasing depth and detail. Currently, thousands of proteins and their post-translational modifications in complex biological samples can be analyzed in parallel. Quantitative techniques, such as stable isotope labeling by amino acids in cell culture (SILAC) and tandem mass tags (TMTs), allow to accurately determine changes in protein abundance over time and between different conditions, even within a single liquid chromatography (LC)–MS run [6,7]. Nevertheless, minor changes in protein abundance are challenging to detect and are often overlooked in bottom-up analyses. Therefore, targeted detection of newly synthesized proteins (NSPs) is a requisite to elucidate minute differences in protein synthesis. Consequently, dedicated MS-based methods have been developed to study protein synthesis (Figure 2).

## Puromycin-based approaches

Puromycin is an aminonucleoside antibiotic, produced by *Streptomyces alboniger*, and has served as inspiration for the development of powerful molecular tools to study NSPs [8]. Puromycin inhibits protein synthesis by ribosome-catalyzed incorporation into the C-terminus of nascent polypeptide chains, preventing further polypeptide chain extension and causing premature termination of protein translation. The development of various puromycin-based reagents with additional features, such as fluorophores, photocaging groups, and radiolabels, has allowed spatiotemporal visualization of protein synthesis with applications both *in vitro* and *in vivo* [8]. In contrast to radiolabeling and fluorescence microscopy, the use of puromycin-based derivatives in combination with MS-based proteomics allows proteome-wide analysis of protein synthesis at an individual protein level resolution.

A biotin conjugate of puromycin has recently been introduced to facilitate puromycin-associated nascent chain proteomics (PUNCH-P) [9,10]. PUNCH-P allowed the detection of thousands of NSPs derived from cells or tissue. Cell cycle–specific fluctuations in protein synthesis in cell lines were monitored, and the so-called ‘translatome’ of a whole mouse brain could be charted [10]. Because puromycin–biotin presents poor cell permeability, ribosome isolation is required before puromycin–biotin labeling. In contrast, the puromycin analog O-propargyl-puromycin (OPP), that is puromycin conjugated with an alkyne, is cell-permeable and thus compatible with live-cell labeling and has been used to analyze NSPs in living early erythroid progenitor cells [11]. OPP was subsequently used in combination with pulsed SILAC (pSILAC), which is the pulsed metabolic incorporation of stable isotope-labeled amino acids in NSPs. The addition of pSILAC allows controlling for the background caused by nonspecific binders during enrichment, providing a more accurate quantification of protein synthesis [12]. The cell-permeable feature of OPP is a significant advantage over PUNCH-P, where subtleties of the cellular context may become lost on ribosome isolation. However, extended *in situ* labeling times with OPP results in the accumulation of truncated puromycin-bound peptides, and inhibition of protein translation can affect the cellular processes under investigation. To alleviate these issues, Tong et al. [13] developed quantitative OPP tagging, a combination of OPP with TMT labeling which enabled a substantial reduction in labeling time, to 15 min, and the quantitation of over 3000 NSPs. Quantitative OPP tagging could track dynamic changes in protein synthesis in THP-1 macrophages after lipopolysaccharide treatment. Photocaged puromycin analogs, which do exist but have not been implemented for MS applications yet, have the potential to provide an additional level of spatial resolution in studying protein synthesis [8].

### Puromycin-based approaches *in vivo*

Although incorporation of puromycin analogs in nascent polypeptide chains is a powerful approach, it can be challenging to apply *in vivo* because prolonged puromycin incubation time causes toxicity in animals because of inhibited translation and truncated puromycinylated peptides. Despite previous success in studying protein synthesis in tissue and hematopoietic stem cells by fluorescence microscopy and western blot, coupling *in vivo* puromycin labeling to MS-based proteomics to study protein synthesis at a proteome-wide level at the resolution of single proteins remains a significant challenge [14, 15, 16].

## BONCAT

Bio-orthogonal noncanonical amino acid tagging (BONCAT) relies on pulsed metabolic incorporation of noncanonical amino acids (NCAAs) in NSPs. Alkyne or azide ligation handles in NCAAs enable bio-orthogonal ligation to reporter groups and subsequent enrichment of NSPs. Multiple *L*-methionine analogs have been used for BONCAT, such as *L*-azidohomoalanine (AHA), *L*-homopropargylglycine (HPG), and *L*-azidonorleucine (ANL) (Figure 2) [17, 18, 19, 20]. From these reagents, AHA is the most used and translationally active with incorporation rates of 400 times lower than methionine, whereas HPG has an incorporation rate that is 500 times lower than methionine, and ANL is not incorporated in wild-type cells and requires expression of a mutant methionine-tRNA synthetase for incorporation [17,19,21].

Since its introduction in 2006 as an MS-based approach to measure protein synthesis, BONCAT has been widely used to investigate protein synthesis in physiological and disease processes. BONCAT has been deployed to analyze protein synthesis in tumor necrosis factor alpha– and interleukin 1 beta–dependent inflammatory response, T cell activation, oxytosis, and in neurons [22, 23, 24, 25, 26, 27]. BONCAT was also used successfully *in vivo* in *Caenorhabditis elegans*, zebrafish, and *Xenopus* [28, 29, 30]. Despite the wide recognition and success of BONCAT-based methods, some challenges remain. About 6% of the proteome is undetectable for BONCAT, as these proteins do not contain any methionine residues or solely a methionine which is directly cleaved after release from the ribosome [20,31]. In addition, enrichment with streptavidin for biotinylated proteins is prone to undesired binders, such as endogenously biotinylated proteins, hampering unambiguous identification of *bona fide* NSPs [32]. Finally, measurement of protein synthesis with high temporal resolution or in challenging systems with low metabolic rates is challenging when using BONCAT because of the often low levels of tagged proteins.

### Direct detection of labeled peptides

To alleviate the problem of falsely identified NSPs due to enrichment of endogenously biotinylated proteins and nonspecific binding to immobilized streptavidin, direct detection of NCAA-containing peptides should be used. Detection of NCAA-labeled peptides can be achieved using desthiobiotin, biotinylation site identification technology, or direct detection of biotin-containing tags, which allow the elution and analysis of (desthio)biotinylated peptides by LC-MS [32, 33, 34]. In addition, various cleavable biotin linkers have been introduced which could directly detect BONCAT-labeled peptides derived from NSPs [20,35]. Alkynylated resin presents a viable option as well [36]. A novel enrichment approach, PhosID, for the enrichment of AHA-labeled peptides was recently reported [37]. The phosphonic acid handle, inspired on an enrichable crosslinker for MS termed PhoX, enables automated Fe^3+^–immobilized metal affinity chromatography enrichment and direct analysis of labeled peptides by LC-MS/MS [38]. A total of 176 NSPs were found to be significantly regulated by treatment of interferon-γ in HeLa cells, of which many had been previously reported to be interferon responsive genes [37].

### Combining BONCAT with stable isotope labeling techniques

Another strategy to differentiate between *bona fide* NSPs and nonspecific binders is the combination of BONCAT and pSILAC, analogous to the combination of OPP and pSILAC [12]. In this strategy, both NCAAs and stable isotope-labeled amino acids are metabolically incorporated in the cell in a pulsed fashion. BONCAT combined with pSILAC labeling, termed quantitative NCAA tagging (QuaNCAT), has been used to reveal alterations in protein synthesis upon T cell activation [23]. In addition, using a combination of pSILAC and BONCAT allowed the study of brain-derived neurotrophic factor–induced protein synthesis in hippocampal mouse brain slices [39]. As an alternative, heavy isotope-labeled AHA quantification was found to be more sensitive than QuaNCAT, as the biotinylated peptides were directly detected by means of the direct detection of biotin-containing tags protocol [24]. The heavy labeled AHA allowed for relative quantification of NSPs expressed during oxytosis in HT22 and HEK293T cells. Of note, combining QuaNCAT with TMT labeling enables highly multiplexed quantitative measurements of protein synthesis across multiple time points (multiplex isobaric tagging/noncanonical amino acid tagging). Using this additional quantitative technique allowed to study the rate of protein synthesis over time upon epidermal growth factor stimulation with a 15 min resolution [40]. Another study combined pSILAC with BONCAT and TMT labeling to investigate the half-lives of NSPs in MCF-7 cells after bortezomib (proteasome inhibitor) or 3-methyladenine (lysosome inhibitor) treatment [41]. These innovations demonstrate that stable isotope labeling techniques combined with BONCAT enable higher sensitivity, temporal resolution, and produce fewer false positives.

### BONCAT in illustrative challenging systems

The high sensitivity of the current mass spectrometers now enables the analysis of NSPs in systems with low metabolic rates and consequently low protein synthesis. A combination of BONCAT with isobaric tags for relative and absolute quantitation was used to monitor protein synthesis in the parasite *Leishmania mexicana* during starvation to uncover the underlying molecular mechanisms facilitating adaptation to stressful conditions [42]. The starvation time-dependent increase of expression of several proteins that potentially play crucial roles in the endoplasmic reticulum stress response pathways in the parasite was identified. These data show that BONCAT is suited to probe the response of parasites to external stimuli and can aid the discovery of new drug targets in parasites. Van Gelder et al. [26] studied protein synthesis dependent on metabotropic glutamate receptor activation in primary hippocampal neurons. It is anticipated that the study of protein synthesis in even more challenging systems is within reach using BONCAT-based approaches, combined with stable isotope labeling, especially as mass spectrometers become even more sensitive.

### Cell type–specific protein synthesis

In contrast to AHA or HPG, labeling with ANL provides the opportunity to retain some spatial information in MS-based NSP analysis, as a prerequisite for the metabolic incorporation of ANL is the (cell-specific) expression of a mutant methionine-tRNA ligase that recognizes ANL. Several studies have used this technique to monitor cell types of interest *in vivo* [43,44]. A tumor-specific proteome was consequently labeled and identified by using ANL *in vivo* [44]. Importantly, with this method, synthesis events in cell types of interest could be studied as well. This has been demonstrated by Alvarez-Castelao et al. [45] and Evans et al. [46] who performed *in vivo* cell type–specific labeling of NSPs in hippocampal neurons in mice. Cell type–specific NSP analysis is the first step toward obtaining spatial resolution in BONCAT experiments, and subcellular expression of tRNA-synthetase mutants might provide further spatial information of synthesis events.

## Dynamic SILAC-based approaches

**Figure 3:**
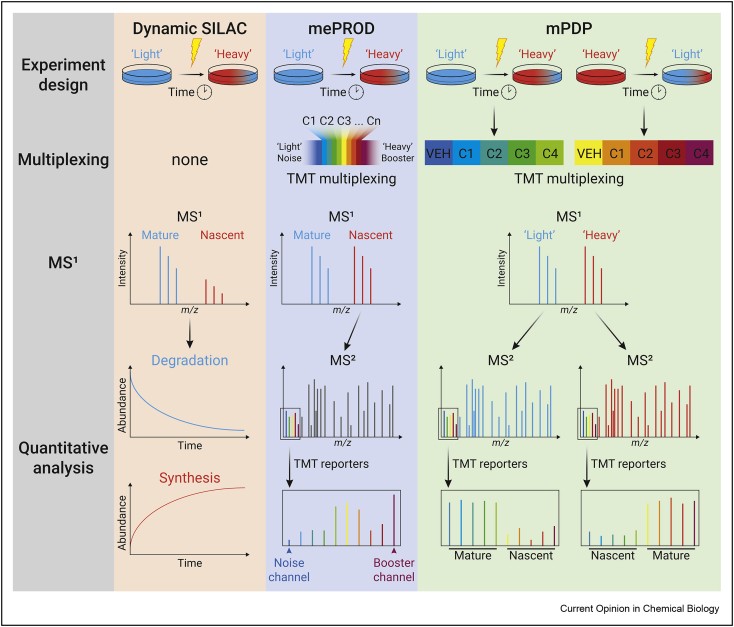
**Dynamic SILAC-based methods**. Dynamic SILAC uses pulsed SILAC to identify isotopically labeled proteins as NSPs, and the decrease of nonlabeled is proportional to protein degradation. Multiplexed enhanced protein dynamics (mePROD) and multiplexed proteome dynamics profiling (mPDP) are both methods that combine TMT multiplexing with dynamic SILAC. mePROD also includes a ‘heavy’ booster and ‘light’ ‘noise’ channel for MS1 triggering and to improve the accuracy of quantification. mPDP creates signal amplification in the MS^1^ channel by using both a ‘light’ to ‘heavy’ and a ‘heavy’ to ‘light’ switch for each condition, resulting in robust detection and quantification of synthesis and degradation by means of the individual TMT channels.

Protein levels are regulated by the interplay of protein synthesis and degradation. Therefore, to obtain a comprehensive view of protein dynamics, it is also important to monitor protein degradation and measure the half-life of individual proteins. Compared with the aforementioned techniques, labeling of cells or organisms using exclusively pSILAC is less invasive to the biological system under investigation. After pulsed labeling of nonlabeled proteomes with isotopically labeled amino acids, labeled proteins can be identified as NSPs, and the concurrent decrease of nonlabeled proteins is proportional to protein degradation. Thereby, pSILAC allows *in vitro* and *in vivo* monitoring of both protein synthesis and degradation simultaneously, termed ‘dynamic SILAC’ (Figure 3). A limitation of this method is the inability to enrich for these isotope-labeled proteins or peptides. Therefore, substantially longer labeling time are required compared to BONCAT- or puromycin-based approaches, and consequently, rapid synthesis and degradation events are challenging to monitor with this method.

Dörrbaum et al. [47] used dynamic SILAC to investigate protein synthesis and degradation in drug-induced homeostatic upscaling or downscaling of primary cultured neurons. The study design included a stable isotope-labeled internal standard, allowing high precision quantification of small changes in protein synthesis and degradation. Consequently, a comprehensive profile of protein dynamics in neurons during upscaling or downscaling was obtained [47]. Furthermore, pSILAC was used to investigate translation of proteins in neuronal injury and consequent regenerative axon regrowth [48]. Of note, pSILAC is challenging in these nondividing cell lines as the labeling was barely sufficient to distinguish between injured cells and the control groups.

### Combining dynamic SILAC with isobaric labeling

Multiplexed enhanced protein dynamics adds multiplexing by TMT labeling to allow the analysis of acute changes in protein synthesis and enables detection of minor differences in protein synthesis after only 2 h in fewer than 100,000 cells. Signal amplification was achieved by the inclusion of an isotopically labeled booster channel for MS1 triggering (Figure 3). Although booster channels enable the identification of more peptides, high levels of booster proteome may adversely affect quantitative accuracy [49]. Multiplexed enhanced protein dynamics was used to study the eIF2alpha- and mTOR-dependent pathways in the integrated stress response. Crosstalk between these two pathways was observed [50]. Savitski et al. used dynamic SILAC combined with TMT labeling, called ‘multiplexed proteome dynamics profiling’, to investigate the effects of estrogen receptor modulators on protein homeostasis in MCF-7 cells, and differential effects on protein synthesis and degradation between various modulators were observed [51,52]. These studies demonstrate the power and potential of combining dynamic SILAC with isobaric labeling for concomitant analysis of protein synthesis and degradation.

## Conclusions

MS-based proteomics in various combinations with stable isotope labeling and affinity enrichment has led to a powerful toolbox to study NSPs. The recent advances in these methodologies, including completely novel enrichment strategies, and the application and combination of multiple (stable isotope-based) quantitative techniques allow the investigation of NSPs with increasing accuracy, temporal, and spatial resolution in ever more challenging systems. Puromycin-based approaches, BONCAT, and pulsed/dynamic SILAC are currently still the core techniques used to study NSPs. These orthogonal techniques all have their own unique strengths and weaknesses, as we aimed to summarize in Table 1. The extensive toolbox available to study NSPs allows researchers the selection of suitable methods for specific research questions. We anticipate that the field will develop further toward methods with an even higher sensitivity, temporal resolution, and spatial resolution and to the level of detail whereby newly synthesized proteoforms can also be analyzed. As studying NSPs is key to understanding changes in the proteome in health and disease and enables the discovery of new therapeutic targets, further development of such tools remains essential.

**Table 1:** Discussed approaches and their characteristics.

Strategy	Method	Invasiveness	Labeling time	Enrichment	Multiplexing	Degradation analysis	Spatial resolution	Reference
Puromycin-based	PUNCH-P	–	15 min	+	No	–	−−	[10]
	OPP	–	2 h	+	No	–	−−	[11]
	OPP-pSILAC	–	2 h	+	No	–	−−	[12]
	QOT	+	15 min	+	Yes	–	−−	[13]
BONCAT-based	AHA/HPG	++	Hrs/days	+	No	+	−−	[17]
	ANL	–	Days (*in vivo*)	+	No	+	+	[43, 44, 45, 46∗]
	QuaNCAT	++	2–4 h	+	No	+	−−	[23]
	MITNCAT	++	15 min	+	Yes	+	−−	[40]
	HILAQ	++	1 h	++	No	+	−−	[24]
	PhosID	++	4–24 h	++	No	+	−−	[37]
SILAC-based	Dynamic SILAC	+++	6 h/days	N/A	No	++	−−	[47,48]
	mPDP	+++	3–48 h	N/A	Yes	++	−−	[50]
	MePROD	+++	2 h	N/A	Yes	++	−−	[49]

QOT, quantitative OPP tagging; MITNCAT, multiplex isobaric tagging/noncanonical amino acid tagging; HILAQ, heavy isotope-labeled AHA quantification; mPDP, multiplexed proteome dynamics profiling; MePROD, multiplexed enhanced protein dynamics.

Beneficial attributes are represented with ‘+’, whereas limitations are indicated as ‘−’. ‘Invasiveness’ describes the adverse effect of the technique on the system under investigation.

## Declaration of competing interest

The authors declare that they have no known competing financial interests or personal relationships that could have appeared to influence the work reported in this paper.
